# Enhancing studies of the connectome in autism using the autism brain imaging data exchange II

**DOI:** 10.1038/sdata.2017.10

**Published:** 2017-03-14

**Authors:** Adriana Di Martino, David O’Connor, Bosi Chen, Kaat Alaerts, Jeffrey S. Anderson, Michal Assaf, Joshua H. Balsters, Leslie Baxter, Anita Beggiato, Sylvie Bernaerts, Laura M. E. Blanken, Susan Y. Bookheimer, B. Blair Braden, Lisa Byrge, F. Xavier Castellanos, Mirella Dapretto, Richard Delorme, Damien A. Fair, Inna Fishman, Jacqueline Fitzgerald, Louise Gallagher, R. Joanne Jao Keehn, Daniel P. Kennedy, Janet E. Lainhart, Beatriz Luna, Stewart H. Mostofsky, Ralph-Axel Müller, Mary Beth Nebel, Joel T. Nigg, Kirsten O’Hearn, Marjorie Solomon, Roberto Toro, Chandan J. Vaidya, Nicole Wenderoth, Tonya White, R. Cameron Craddock, Catherine Lord, Bennett Leventhal, Michael P. Milham

**Affiliations:** 1 The Child Study Center at NYU Langone Medical Center, New York, New York 10016, USA; 2 Child Mind Institute, New York, New York 10022, USA; 3 Nathan S. Kline Institute for Psychiatric Research, Orangeburg, New York, New York 10962, USA; 4 KU Leuven, Department of Rehabilitation Sciences, Neuromotor Rehabilitation Research Group 3000, Leuven, Belgium; 5 Division of Neuroradiology, University of Utah, Salt Lake City, Utah 84112, USA; 6 Interdepartmental Program in Neuroscience, University of Utah, Salt Lake City, Utah 84116, USA; 7 The Brain Institute at the University of Utah, Salt Lake City, Utah 84116, USA; 8 Department of Bioengineering, University of Utah, Salt Lake City, Utah 84116, USA; 9 Olin Neuropsychiatry Research Center, The Institute of Living, Hartford Hospital, Hartford, Connecticut 06102, USA; 10 Department of Psychiatry, Yale University School of Medicine, New Haven, Connecticut 06510, USA; 11 Neural Control of Movement Lab, ETH Zürich, Zürich 8092, Switzerland; 12 Neuroimaging Research, Barrow Neurological Institute, Phoenix, Arizona 85013, USA; 13 Department of Child and Adolescent Psychiatry, Robert Debré Hospital, APHP, 75019 Paris, France; 14 Human Genetics and Cognitive Functions, Institut Pasteur, 75015 Paris, France; 15 Department of Child and Adolescent Psychiatry, Erasmus University Medical Centre, 3015 Rotterdam, Netherlands; 16 Center for Cognitive Neuroscience, UCLA, Los Angeles, California 90095, USA; 17 Department of Psychiatry & Biobehavioral Science, Semel Institute for Neuroscience and Human Behavior, UCLA, Los Angeles, California 90095, USA; 18 Interdepartmental Neuroscience Program, UCLA, Los Angeles, California 90095, USA; 19 David Geffen School of Medicine, UCLA, Los Angeles, California 90095, USA; 20 Department of Speech and Hearing Science, Arizona State University, Tempe, Arizona 85004, USA; 21 Department of Psychological and Brain Sciences, Indiana University, Bloomington, Indiana 47405, USA; 22 Ahmanson-Lovelace Brain Mapping Center, UCLA, Los Angeles, California 90095, USA; 23 Behavioral Neuroscience Department, Oregon Health & Science University, Portland, Oregon 97239, USA; 24 Psychiatry Department, Oregon Health & Science University, Portland, Oregon 97239, USA; 25 Advanced Imaging Research Center, Oregon Health & Science University, Portland, Oregon 97239, USA; 26 Department of Psychology, San Diego State University, San Diego, California 92182, USA; 27 Department of Psychiatry, School of Medicine, Trinity Centre for Health Science, St James’s Hospital, Dublin 8, Ireland; 28 Trinity Institute of Neuroscience, Trinity College Dublin, Dublin 2, Ireland; 29 Department of Psychiatry, University of Utah, Salt Lake City, Utah 84108, USA; 30 Waisman Center, University of Wisconsin-Madison, Madison, Wisconsin 53705, USA; 31 University of Pittsburgh School of Medicine, Pittsburgh, Pennsylvania 15261, USA; 32 Center for Neurodevelopmental and Imaging Research, Kennedy Krieger Institute, Baltimore, Maryland 21205, USA; 33 Department of Neurology, Johns Hopkins School of Medicine, Baltimore, Maryland 21205, USA; 34 Department of Psychiatry, Johns Hopkins School of Medicine, Baltimore, Maryland 21205, USA; 35 UC Davis Department of Psychiatry and Behavioral Science, California 95817, USA; 36 UC Davis MIND Institute, Sacramento, California 95817, USA; 37 Children’s Research Institute, Children’s National Medical Center, Washington, District Of Columbia 20010, USA; 38 Department of Psychology, Georgetown University, Washington, District Of Columbia 20010, USA; 39 Center for Autism and the Developing Brain, Weill Cornell Medical College, White Plains, New York 10605, USA; 40 Department of Psychiatry, University of California San Francisco, San Francisco, California 94103, USA

**Keywords:** Neuroscience, Functional magnetic resonance imaging, Magnetic resonance imaging, Autism spectrum disorders, Brain imaging

## Abstract

The second iteration of the Autism Brain Imaging Data Exchange (ABIDE II) aims to enhance the scope of brain connectomics research in Autism Spectrum Disorder (ASD). Consistent with the initial ABIDE effort (ABIDE I), that released 1112 datasets in 2012, this new multisite open-data resource is an aggregate of resting state functional magnetic resonance imaging (MRI) and corresponding structural MRI and phenotypic datasets. ABIDE II includes datasets from an additional 487 individuals with ASD and 557 controls previously collected across 16 international institutions. The combination of ABIDE I and ABIDE II provides investigators with 2156 unique cross-sectional datasets allowing selection of samples for discovery and/or replication. This sample size can also facilitate the identification of neurobiological subgroups, as well as preliminary examinations of sex differences in ASD. Additionally, ABIDE II includes a range of psychiatric variables to inform our understanding of the neural correlates of co-occurring psychopathology; 284 diffusion imaging datasets are also included. It is anticipated that these enhancements will contribute to unraveling key sources of ASD heterogeneity.

## Background & Summary

Multiple sources of evidence have substantiated models of abnormal neural connectivity in autism spectrum disorder (ASD)^1–5^. At the macroscale, abnormal connections among brain regions have been revealed by functional and structural neuroimaging in children, adolescents and, adults with ASD^1,6–9^. Yet, both the complexity of the brain connectome^10,11^ and the striking heterogeneity of ASD^12–16^ have hampered efforts to specify the nature of putative dysconnections. In response, open-data sharing is increasingly being encouraged to rapidly amass the large-scale datasets needed to confront heterogeneity, engage a broader range of scientific disciplines, and facilitate independent replications^17–21^. To bring the open data sharing model to autism neuroimaging, the Autism Brain Imaging Data Exchange (ABIDE)^22^ was launched in 2012. The initial ABIDE initiative—now termed ABIDE I—was the first open-access brain imaging repository of resting state functional magnetic resonance imaging (R-fMRI) and corresponding structural data of individuals with ASD and typical controls (*N*=539 and 573, respectively) aggregated from multiple international institutions. Here, we introduce ABIDE II (Data Citation 1), a new multi-site open data resource containing 1,044 independent datasets (ASD *N*=487; Controls *N*=557) created to enhance the significance of the questions that can be addressed regarding the neural correlates of ASD and accelerate the pace of discovery.

The initial ABIDE I effort established the feasibility of aggregating multisite data without prior harmonization, leading to more than 55 peer-reviewed studies in the 48 months since inception. Despite its success, ABIDE I is limited in regard to sample characterization and sample size. Specifically, despite containing more than 1,000 datasets, ABIDE I was not sufficiently large to furnish optimally sized discovery and replication subsamples. By combining the ABIDE I and ABIDE II data resources, investigators can select larger samples for discovery and replication, depending on their investigative endeavors. Replication samples are needed to minimize false positives and avoid settling for ‘approximate replications’^19^—a practice that has plagued biological psychiatry^19^ and neuroscience more broadly^17^. Additionally, as recently demonstrated, the utility of datasets for prediction increases with sample size—even if heterogeneous data sources are used to amass large samples^23^.

Along with increased sample size, ABIDE II provides greater phenotypic characterization than was available across the ABIDE I data collections to better address two key sources of heterogeneity. The first is psychopathology co-occurring with ASD, which has been largely overlooked in the imaging literature^15,16,24^. Accordingly, ABIDE II actively encouraged investigators to provide phenotypic information regarding co-occurring illness, if assessed. The second source of heterogeneity is driven by sex-related differences. These have been generally ignored in the ASD imaging literature due to the markedly higher prevalence of males with ASD and the tendency of single sites to exclude or minimally represent females. The ABIDE II sample has increased the number of available datasets from females with ASD from 65 in ABIDE I to 138 when ABIDE I+II are combined. We believe these enhancements will allow investigators to more directly investigate pathophysiology specific to ASD, to potentially identify neurobiological subgroups and facilitate the identification of protective and risk factors.

Finally, beyond its focus on intrinsic functional connectivity and other indices of intrinsic brain function, ABIDE II now includes a subset of datasets (*N*=284) with diffusion-weighted images. In order to facilitate immediate access and use of ABIDE II, the methods utilized to generate this resource, the resulting currently available data and their technical validation are described below.

## Methods

### Criteria for data contributions

We solicited investigators willing and able to openly share their previously collected awake R-fMRI data of individuals with ASD and controls, along with corresponding high-resolution anatomical images and phenotypic information. Contributions have been sought from all charter ABIDE I members and invitations are extended to any other investigators involved in ASD neuroimaging. The present work includes information regarding all contributions received prior to June 24, 2016. Contributions will continue to be accepted up to December 2016.

Contributors are encouraged to share at least 20 unique datasets per diagnostic group (i.e., ASD and controls). Data collections of only individuals with ASD are also accepted as they can be utilized for data-driven explorations addressing heterogeneity e.g., refs 25,26. Consistent with prior FCP/INDI efforts^27^, investigators are also encouraged to contribute nearly all MRI datasets, without a priori quality criteria (see Technical Validation for quality assessment (QA) measures incorporated into ABIDE II).

The availability of minimal phenotypic information essential for data analyses and sample characterization (i.e., diagnostic classification, age, sex) is required for contribution. To enhance phenotypic characterization, sharing of additional measures commonly used in ASD research, information on psychiatric comorbidity, medication status, cognition and/or language are highly encouraged. Similarly, to enhance the breath of investigations about the ASD connectome, whenever available, contributions of corresponding diffusion images for each individual are welcome for aggregation.

Finally, prior to data contribution, sites are required to confirm that their local Institutional Review Board (IRB) or ethics committee have approved both the initial data collection and the retrospective sharing of a fully de-identified version of the datasets (i.e., after removal of the 18 protected health information identifiers including facial information from structural images as identified by the Health Insurance Portable and Accountability Act [HIPAA]).

Of note, two institutions provided longitudinal MRI scans from subsets of individuals’ datasets (*n*=23 ASD and *n*=15 controls) previously contributed to ABIDE I. Given the relevance of developmental changes^28–32^, these datasets are also included in the ABIDE II. To distinguish them from the cross-sectional aggregates, these datasets are organized into a separate set of collections focused on longitudinal data using the original ABIDE I IDs.

### Data preparation and aggregation

Prior to contribution, each institution is asked to rename all data by replacing local subject identification numbers with FCP/INDI identifiers. They are also asked to remove personally identifying information (PHI) including those from images (e.g., NIFTI headers and face information from any high-resolution images) using the FCP/INDI anonymization script available in http://fcon_1000.projects.nitrc.org/. Once data are fully anonymized at each site, they are submitted to the coordinating centers (Nathan Kline Institute and New York University) for review and harmonization within and across sites. Specifically, MRI data are visually inspected and edited as needed to ensure complete removal of facial information. Additionally, to further protect personal privacy, images of ears are removed from high-resolution images. Regarding phenotypic datasets, each entry is also reviewed to identify and correct missing data, any impossible entry values (e.g., beyond published maxima and minima), and extreme outliers (relative to each sample). To ensure uniformity across sites, all entries are recorded as needed and organized in a common template along with a legend of code keys. As a final step in preparation for release, both donating and coordinating sites jointly prepare a narrative for each data collection, documenting information on the methods utilized, funding sources, the investigators involved, whether any link with other databases (e.g., National Database for Autism Research—NDAR^33^) exists, along with publications related to the contributed datasets. Before open release, each donating site reviews their reorganized phenotypic records, five random images per imaging modality and their collection-specific narrative for final approval.

## Data Records

### Overview

**Figure 1:**
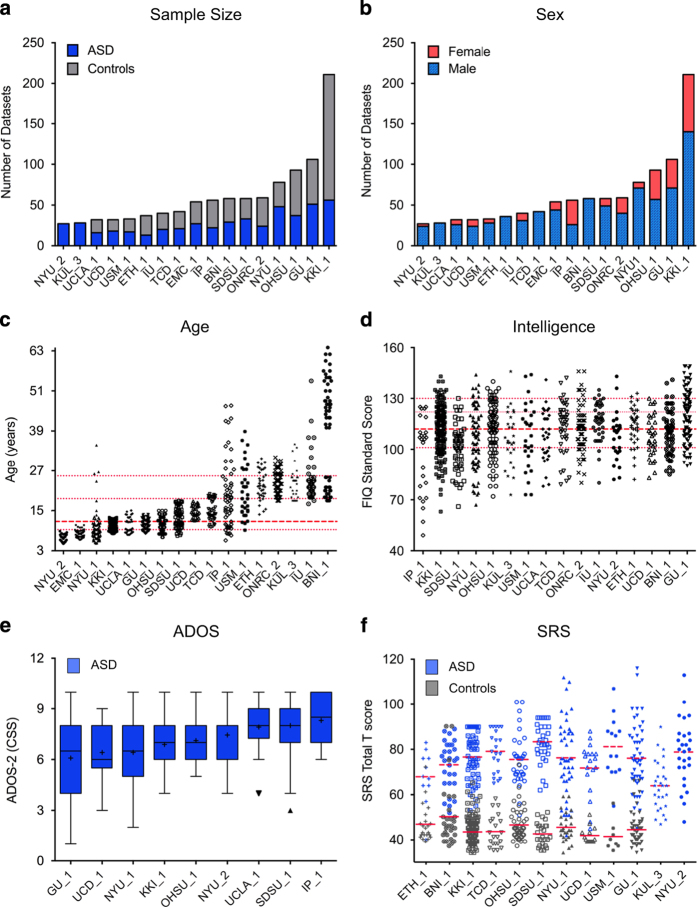
Key phenotypic characteristics. (**a**) Total number of datasets per group (gray=controls; blue=autism spectrum disorder (ASD)) for the 17 cross-sectional ABIDE II data collections (i.e., collections from individuals not included in ABIDE I). Data are ordered as a function of sample size. (**b**) Number of males (light blue) and females (red) for each data collection, irrespective of diagnostic group. Data are ordered as a function of sample size. (**c**) Age at time of scan in years per collection (ordered by mean age per collection), irrespective of diagnostic group. The median age across collections (11.7 years) is depicted with a thick red dashed line; 25th, 75th, and 90th percentiles (9.3, 18.6, and 25.5 years, respectively) are represented by thin red dashed lines. (**d**) Distribution of full scale IQ (FIQ) standard scores per collection (ordered by lowest FIQ included per collection) for all datasets, irrespective of diagnostic group. The median FIQ across collections (112) is depicted with a thick red dashed line; 25th, 75th, and 90th percentiles (101, 122, and 130, respectively) are represented by thin red dashed lines. (**e**) Tukey’s box-whiskers plots depict the distribution of Autism Diagnostic Observation Schedule, Second Edition (ADOS-2) total calibrated severity scores (CSS) for ASD datasets in the nine collections sharing them (ordered by mean CSS per collection). The black plus sign depicts the mean CSS for each collection. (**f**) Distribution of Social Responsiveness Scale (SRS) total T scores (gray=controls; blue=ASD) in the 12 collections sharing them. For each collection, red dashed and solid lines indicate mean SRS total T scores of ASD and controls, respectively.

The current ABIDE II dataset encompasses 17 collections of unique independent datasets (i.e., from individuals whose data were not previously shared in ABIDE I) yielding 487 datasets classified as ASD and 557 as controls (Fig. 1a, Table 1). These represent previously collected datasets across 16 sites, including nine charter ABIDE I institutions and seven new members (See Supplementary Table 1 for information on each institution). A simple naming convention is used to label each data collection: <ABIDEII> -<institution acronym name>_<collection number>(e.g., ABIDEII-NYU_1). When a collection in ABIDE II is a continuation of one initiated in ABIDE I, we employ the same collection number used in ABIDE I (or 1 if none was used, e.g., SDSU_1, KKI_1). For new collections, a unique consecutive number is assigned (e.g., BNI_1, KUL_3). Accompanying the primary cross-sectional aggregate, two longitudinal collections are also aggregated in ABIDE II. These include MRI datasets collected as follow-ups to the MRI and phenotypic data released in ABIDE I (N total=38 unique IDs). These pilot longitudinal collections are identified as <ABIDEII> -<institution acronym name>_<Long> (Table 1).

All ABIDE II datasets can be accessed, after establishing a login and user password, through FCP/INDI at the Neuroimaging Informatics Tools and Resources Clearinghouse (NITRC; http://fcon_1000.projects.nitrc.org/indi/abide/). The datasets are organized by data collection and stored in.tar files, each containing imaging and phenotypic data.

### Phenotypic information

**Figure 2:**
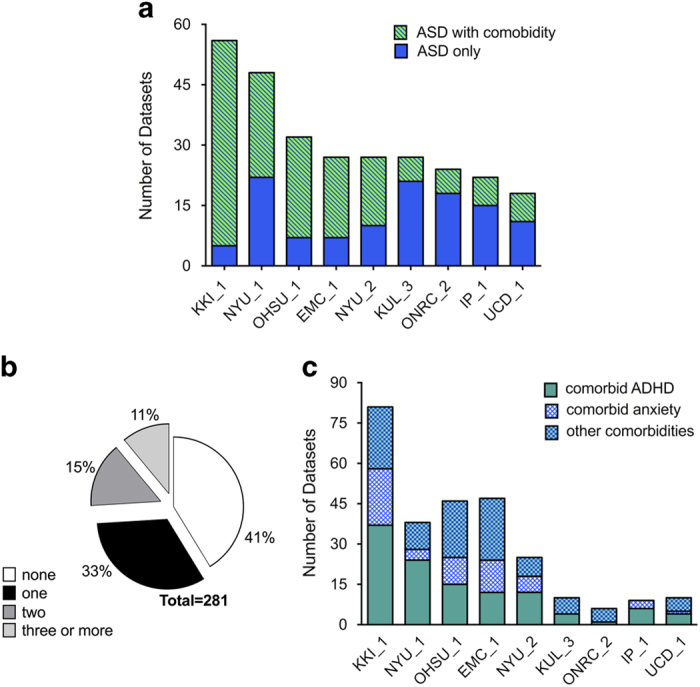
Data on psychiatric comorbidity in Autism Spectrum Disorder (ASD). (**a**) Number of ASD datasets with and without psychiatric diagnoses for each of the nine data collections sharing information on categorical psychiatric diagnoses other than ASD. (**b**) Percentage of ASD datasets with one (black), two (dark gray) or more (light gray) comorbid diagnoses and those without any comorbidity (white) across the nine collections sharing comorbidity information. (**c**) Distribution of ASD datasets with comorbidity divided into those with comorbid Attention-Deficit/Hyperactivity Disorder (ADHD, green), anxiety (blue/white pattern) or others (cyan-white pattern) for each collection. Other comorbidities include enuresis, and/or mood, speech and language and/or disruptive behavior disorders. Here, given that multiple comorbid disorders can co-occur, the number of comorbid ASD datasets across categories exceeds the absolute number of comorbid ASD datasets.

All phenotypic data are stored in comma separated value (.csv) files. A legend describing each phenotypic variable source is available at the website http://fcon_1000.projects.nitrc.org/indi/abide/abide_II.html. Phenotypic files are organized by data collection; a phenotypic composite file including all variables across all collections is also available. Counts of phenotypic variables available for each collection and distributions of selected key variables for each diagnostic group are provided in Supplementary Tables 2 and 3. Below, we briefly describe the main demographics and key phenotypic variables provided in the 17 cross-sectional ABIDE II data collections (Figs 1 and 2).

#### Diagnostic classification

A dummy variable indicates diagnostic group (1 and 2 for ASD and controls, respectively). Given the retrospective nature of this data aggregate, assessment protocols used to identify ASD and controls varied across institutions. They are documented in each data collection narrative. Briefly, ASD classification was determined by either 1) combining clinical judgment with ‘gold standard’ diagnostic instruments—Autism Diagnostic Observation Scale^34,35^ and/or Autism Diagnostic Interview-Revised^36^ [ADOS, ADI-R]; (*n*=12 data collections; 368 ASD datasets) or 2) by using these ‘gold standard’ diagnostic instruments only (*n*=4 collections; 92 ASD datasets), with one exception. Specifically, in EMC_1 (*n*=27 datasets), which was selected from the longitudinal Generation R sample^37^, the ASD classification was based on prior medical records documenting ASD among those individuals meeting screening cutoffs in at least one of two distinct ASD questionnaires or for whom the mother reported a diagnosis of ASD. Regarding controls (*N*=557, available for 15 collections), all datasets are characterized by absence of ASD diagnosis and absence of history of any other major neurodevelopmental disorders for the vast majority of the datasets (*N*=546; 98%). This was determined using semi-structured/unstructured in-person interviews (*N*=7 data collections; 353 datasets), or parent/self- (if adults) reports/questionnaires (*N*=8; 193 datasets). The remaining 11 control datasets (OHSU_1 data collection) are from individuals assigned a ‘rule out’ psychiatric disorder, but without ASD or Attention-Deficit/Hyperactivity Disorder (ADHD) diagnoses.

Other specific inclusion/exclusion criteria used for selecting controls (e.g., IQ range, first degree relative with ASD) or ASD (e.g., absence of reported seizure and genetic syndromes) varied across collections. Each collection narrative on the ABIDE II website provides details regarding these criteria.

#### Demographics

Across collections, age at time of scanning ranges from 5 to 64 years; four of the collections focused specifically on adults—with one of these collections specifically enrolling on older adults (BNI_1)—and eight enrolling only children and/or adolescents. The remaining five data collections include children, teens and young adults, which allows for cross-sectional age-related explorations (Fig. 1c; Supplementary Table 3). All but four collections include data from both sexes (Fig. 1b). Reflecting the higher prevalence of males in ASD^38^, 15% of the ASD datasets consist of females versus 31% of the control datasets (Supplementary Table 3).

#### Intelligence

Full scale intelligence quotient (FIQ) and/or verbal and/or performance IQ standard scores are provided. Across collections, although variation exists with respect to the minimum FIQ, 97% of the datasets have FIQ above 80 (Fig. 1d). For both groups, mean FIQ is above average, albeit significantly higher in controls versus ASD (Mann Whitney U=86.5; *P*<0.0001; Supplementary Table 3).

#### Handedness

Categorical handedness codes for right, left or mixed handedness are available across all collections. Additionally, handedness strength scores are available for eight collections, enabling dimensional characterization of handedness (*n*=244 ASD and *n*=327 controls). Across collections, right-handedness is more frequent in both diagnostic groups (84 and 90% for ASD and controls, respectively), though a significantly higher prevalence of non-right-handedness (either left or mixed handedness) occurs in ASD relative to controls (χ_1_^2^=10.6, *P*=0.01; Supplementary Table 3).

#### ASD core measures

Scores from the ADOS and ADI-R are available (Supplementary Table 3). Only nine collections share ADOS-2 calibrated severity scores^34^ (CSS; *N*=9 sites; 228 ASD datasets) recently designed to adjust for differences in age, intellectual abilities and language skills across ADOS modules^39,40^. As illustrated in Fig. 1f, CSS distribution are similar across most sites. ADOS-G^41^ scaled total scores are available for 15 collections (*n*=280 ASD datasets). Additionally, data from parent or self-report questionnaires commonly used in the field to quantify severity on multiple ASD domains collected across both diagnostic groups are also available. The Social Responsiveness Scale^42^ is the most common (*n*=378 ASD, *n*=407 controls; Fig. 1f) followed by the Repetitive Behavior Scale Revised^43,44^ (*n*=217 ASD, *n*=208 controls; Supplementary Table 3).

#### Comorbid psychopathology in ASD

Information on psychopathology accompanying ASD is provided either as 1) categorical diagnostic labels (or its absence, if assessed) with corresponding diagnostic code based on the International Classification of Diseases-9th edition^45^ (*N*=9 data collections; 281 ASD datasets) and/or as severity scores in one or multiple psychopathology dimensions across available for 11 collections (see Supplementary Table 3 for a list of measures used) (Fig. 2). Categorical comorbid psychiatric diagnoses were determined based on clinicians’ assessments in seven data collections, parent-questionnaires in one data collection (UCD_1) and self-report in another (KUL_3). Consistent with the clinical literature^46–48^, approximately 60% of the ASD data correspond to individuals with one or more co-occurring psychiatric diagnoses (Fig. 2b); the most frequent are ADHD and anxiety disorders.

### MRI data

For each of the 17 ABIDE II cross-sectional collections, for each unique ID#, at least one structural MRI (sMRI), one corresponding R-fMRI dataset are available (except for one individual in the IP collection for which only MRI is available); corresponding diffusion MRI (dMRI) datasets are available for six collections. One data collection (SDSU) provided field map-corrected version of its R-fMRI and DTI data. The two pilot longitudinal collections include sMRI and R-fMRI datasets collected at two time points (1–2 years apart) for 23 individuals with ASD and 15 controls.

Consistent with its popularity in the imaging community and prior usage in FCP/INDI efforts, the NIFTI file format was selected for storage of the ABIDE II MRI datasets. With the exception of a single collection (IP_1, 1.5 Tesla), all MRI data were acquired using 3 Tesla scanners. Table 1 lists the specific MRI scanners and head coils utilized for each collection, along with the number of individuals available for each MRI modality within diagnostic groups (i.e., ASD and controls). Specific MRI sequence parameters for the various data collections are summarized in Tables 2, 3, 4 and detailed on the ABIDE II website. Across collections, R-fMRI acquisition durations varied from five to eight minutes (6:21±0.04 min) per individual; in all but four collections, individuals were verbally asked to keep their eyes open. For 12 collections, exposure to scan simulators prior to scanning was also used for habituation, as documented in the narratives.

## Technical Validation

Consistent with the established FCP/INDI policy, all data contributed to ABIDE II was made available to users regardless of data quality^27^. The rationale of this decision includes the lack of consensus on optimal quality criteria in regards to specific measures or their combinations and cutoffs. Additionally, depending on the study goal, the availability of scans with a range of quality can facilitate the development of artifact correction techniques^18^. For initiatives focusing on clinical populations like ABIDE II, the inclusion of datasets with artifacts such as motion are valuable, as they enable investigators to determine impact of such real-world confounds on reliability and reproducibility.

**Figure 3:**
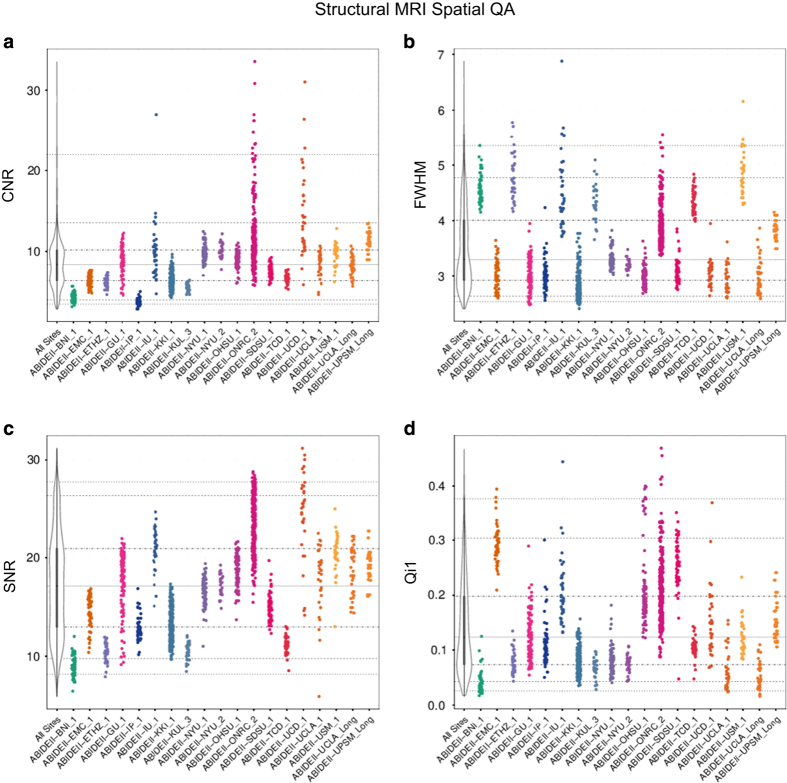
Selection of spatial quality assurance (QA) metrics for high resolution MRI datasets. (**a**) Contrast-to-noise ratio (CNR)^50^, (**b**) smoothness of voxels indexed as full half-width maximum (FHWM)^62^, (**c**) signal-to-noise ratio (SNR)^50^, (**d**) artifactual voxel detection (Q_i_1)^51^- See Table 5 for details on this and the other quality metrics released. The colored scatterplots illustrate the quality metrics distribution for spatial MRI dataset within a given ADBIE II collection (17 cross-sectional and 2 longitudinal collections). The black and white violin plots represent a kernel density estimation of the distribution across all datasets for each quality metrics. The midline thick gray line represents the value that occurs most commonly in the distribution. For each plot the horizontal gray lines mark the 1st, 5th, 25th, 50th (solid gray line), 75th, 95th and 99th percentiles starting from the bottom.

**Figure 4:**
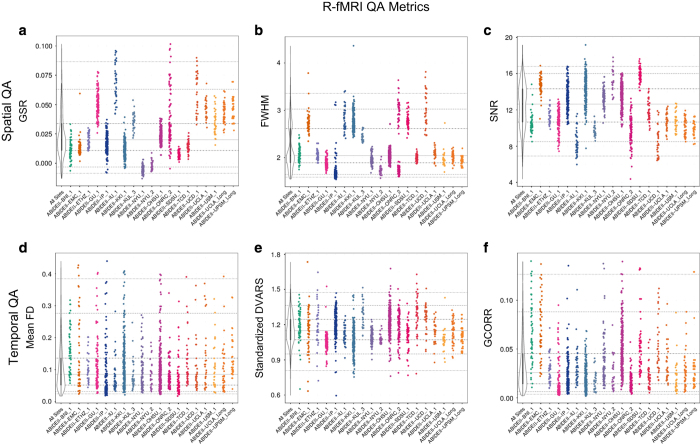
Selection of spatial and temporal quality metrics for resting state functional MRI (R-fMRI). Spatial metrics include: (**a**) Ghost to single ratio (GSR)^50^; (**b**) smoothness of voxels indexed as full-width half maximum (FWHM)^62^, (**c**) signal to noise ratio (SNR)^50^. Temporal metrics are: (**d**) mean framewise displacement^52^; (**e**) standardized DVARS^63^, and (**f**) global correlation (GCORR)^64^—See Table 5 for details on this and the other quality metrics released. The colored scatterplots illustrate the quality metrics distribution for spatial MRI dataset within a given ADBIE II collection (17 cross-sectional and 2 longitudinal collections). The black and white violin plots represent a kernel density estimation of the distribution across all datasets for each quality metrics with its midline thick gray line representing the value that occurs most commonly in the distribution. For each plot, the horizontal gray lines mark the 1st, 5th, 25th, 50th (solid gray line), 75th, 95th and 99th percentiles starting from the bottom.

To facilitate quality assessment of the ABIDE II collections and selection of datasets for analyses by individual users, we used the Preprocessed Connectome Project quality assurance protocol^49^ (http://preprocessed-connectomes-project.github.io). These encompass quantitative metrics commonly used in the imaging literature for assessing data quality, particularly for multisite projects, e.g., ref. 50. They include spatial metrics of scanner performance such as contrast to noise ratio^50^ artifactual voxel detection^51^ as well as temporal metrics including those quantifying head motion^52^; all metrics are summarized in Table 5 and all are available in the data release. As expected by design, within- and between-site variation exists across quality metrics (see Figs 3 and 4 and Supplementary Fig. 1 for examples of spatial and temporal metrics in sMRI, R-fMRI and DTI). It is important to note that the field remains without consensus standards for the usage of QA measures. Additionally, differences in some measures across collections may reflect purposeful tradeoffs in the design of an imaging protocol, which may not be readily obvious at times. As such, caution should be taken in over-interpretation of between-collection differences in QA measures. At a minimum, the various QA measures provided can be used to find outlier datasets for a given site; though, potentially they may be used to provide insights into the impact of differences in acquisition protocols on quality measures as well.

## Usage Notes

As data aggregation followed independent data collections across multiple sites, various sources of heterogeneity exist between collections. They can range from inclusion/exclusion criteria, recruitment/sampling strategies, MRI scanner types, data acquisition parameters and instructions (e.g., eyes open versus closed). Users must be aware of such factors when designing their research questions and selecting data for analyses accordingly. Care should be taken when attempting to draw comparisons across ABIDE I and ABIDE II, as they are independently created aggregate datasets, bringing with them both commonalities and differences. Nine institutions participated in both initiatives with either related collections in regard to both phenotypic and imaging protocols (e.g., NYU_1 in ABIDE II is a continuation of NYU in ABIDE I) or collections acquired through independent protocols (e.g., KUL_3 in ABIDE II). We suggest consideration of the commonalities and differences among contributions when attempting to combine datasets from the two ABIDE initiatives. The narratives included in the ABIDE II website should facilitate this process—see Supplementary Fig. 2 for the collections distributed among the ABIDE initiatives. As a general rule, for aggregate data analyses datasets should be selected to ensure that the number ASD and TDC data are balanced at each collection, unbalanced designs (e.g., all typical participants selected from one collection, all ASD selected from another) should be avoided.

The impact of known and unknown sources of heterogeneity between collections should also be taken in account at the analytical level. First, we encourage the use of standardization at individual- and group-level analyses e.g., refs 53–55. Second, we recommend to model data collection as a covariate at the group level when possible, to account for the variance related to the specific site protocol e.g., refs 53,56,57. Users can also employ meta-analytic approaches that have been shown to be fruitful for examination of cortical thickness or structural volumes e.g., ref. 58. Awareness of site-related variability should also be reflected in the presentation of findings. For example, effects within each data collection should be reported along with those obtained across collections e.g., refs 29,56,59. Inconsistencies that arise may be informative and provide insights into known or unknown differences in samples including and beyond data acquisition protocols. Finally, we note that along with the challenges related to its multisite post-hoc data aggregation, ABIDE II also offers a unique opportunity to develop analytical approaches to address these challenges. For example, a recent effort based on ABIDE I demonstrated the ability to optimize classifiers for the prediction of data from previously unseen imaging sites^23^.

The need for careful consideration of variation in acquisition parameters also applies to the use of the quality assurance (QA) metrics available for the ABIDE-II sample. Some QA measures may be more or less comparable across data collections. Mean FD is an example of a measure commonly used for QA in resting state fMRI studies, albeit without significant considerations on the impact of the specific acquisition protocol employed. Motion-induced fluctuations in the BOLD signal are primarily due to spin history effects and partial voluming, which are proportional to the amount of tissue displacement between subsequent excitations. From this perspective, one might expect that factors capable of impacting spin history effects or partial voluming, would in turn impact meanFD. Importantly, these relationships may not necessarily be linear or additive. As such, some caution is suggested when interpreting systematic differences in meanFD, or related motion metrics (e.g., DVARS), across collections. Users may also employ this and other shared multisite datasets e.g., refs 60,61 to explore the impact of possible differences related to acquisition parameters, such as TR and other, on motion metrics. MeanFD computed in DTI data should not be used for comparisons between different collections with different MRI protocols. Mean FD in DTI is the result of the combination of both eddy current effects and head motion. As a result, meanFD can be used to compare and select data within collections obtained with the same scanning protocols and equipment.

Finally, to facilitate replications among studies using ABIDE data, we encourage users to provide the ID list utilized for their published manuscripts in the manuscript section of the ABIDE website (http://fcon_1000.projects.nitrc.org/indi/abide/manuscripts.html). Users are also requested to acknowledge the primary funding source for ABIDE II (NIMH 5R21MH107045) in any manuscripts using the ABIDE II data.

## Additional Information

**How to cite this article:** Di Martino, A. *et al.* Enhancing studies of the connectome in autism using the autism brain imaging data exchange II. *Sci. Data* 4:170010 doi: 10.1038/sdata.2017.10 (2017).

**Publisher’s note:** Springer Nature remains neutral with regard to jurisdictional claims in published maps and institutional affiliations.

## Supplementary Material



Supplementary Information

## Figures and Tables

**Table 1:** Information on scanners, head coils (same across MRI modalities) and MRI individual's datasets counts for each collection included in the primary ABIDE II collections (i.e., datasets collected in a given institution from individuals not included in ABIDE I) and in the longitudinal pilot collections (i.e., data from the same individuals scanned twice: Time 1 data originally released in ABIDE I and Time 2 data released in ABIDE II).

**Data collection**	**MRI scanner**	**Head coil**			**R-fMRI**		**High res MRI**		**DWI** *	
	**Manufacturer**	**Model**	**T**	**# channels**	***ASD***	***Contr***	***ASD***	***Contr***	***ASD***	***Contr***
*Primary Cross Sectional Aggregate*										
ABIDEII-BNI_1	Philips	Ingenia	3	15	29	29	29	29	29	29
ABIDEII-EMC_1	GE	MR750	3	8	27	27	27	27	0	0
ABIDEII-ETH_1	Philips	Achieva	3	32	13	24	13	24	0	0
ABIDEII-GU_1	Siemens	TriTim	3	12	51	55	51	55	0	0
ABIDEII-IU_1	Siemens	TriTim	3	32	20	20	20	20	0	0
ABIDEII-IP_1	Philips	Achieva	1.5	8	22	33†	22	34	21	32
ABIDEII-KKI_1	Philips	Achieva	3	8‡	56	155	56	155	0	0
ABIDEII-KUL_3	Philips	Achieva Ds	3	32	28	0	28	0	0	0
ABIDEII-NYU_1	Siemens	Allegra	3	8	48	30	48	30	33	24
ABIDEII-NYU_2	Siemens	Allegra	3	8	27	0	27	0	19	0
ABIDEII-ONRC_2	Siemens	Skyra	3	32	24	35	24	35	0	0
ABIDEII-OHSU_1	Siemens	TriTim	3	12	37	56	37	56	0	0
ABIDEII-SDSU_1§	GE	MR750	3	8	33	25	33	25	33	24
ABIDEII-TCD_1	Philips	Achieva	3	8	21	21	21	21	20	20
ABIDEII-UCD_1	Siemens	TriTim	3	32	18	14	18	14	0	0
ABIDEII-UCLA_1	Siemens	TriTim	3	12	16	16	16	16	0	0
ABIDEII-USM_1	Siemens	TriTim	3	12	17	16	17	16	0	0
Total N	487	523	487	557	155	129				
										
*Pilot Longitudinal Aggregate*										
UCLA_Long	Siemens	TriTim	3	12	9	8	14	7	NA	NA
UPSM_Long	Siemens	Allegra	3	8	14	7	9	8	NA	NA
Total N	23	15	23	15	—	—				

*For longitudinal collections Diffusion Weighted Imaging (DWI) data were not included ABIDE I, thus are not applicable (NA) here.

^†^ For IP one individual ID only has sMRI available.

^‡^ For KKI_1 an 8-channel head coil was used for *n*=149 datasets and a 32-channel head coil was used for *n*=62 datasets—a list of the IDs with the corresponding head coil is provided in the ABIDE II website page for this collection under ‘Additional scan Information’.

^§^ SDSU also includes field maps corresponding to the R-fMRI and all DTI datasets but two. ASD, Autism Spectrum Disorder; Contr, Controls; GE, General Electrics; High res MRI, High Resolution Magnetic Resonance Imaging; Res, Resolution; R-fMRI, Resting-state functional MRI; T, Tesla; #, number. Also See Supplementary Table 1 for a list of the institutions/investigators.

**Table 2:** Sequence parameters of structural MRI datasets at each data collection.

**Collection**	**FA**	**TI**	**TE**	**ES**	**BW**	**TR**	**PA**	**PF**	**SO**	**PE**	**Reconstructed Resolution (mm)**			**Recontructed Image Dims**			**TA**
**Label**	**°**	**ms**	**ms**	**ms**	**Hz/Px**	**ms**					**RO**	**PE**	**SL**	**RO**	**PE**	**SL**	**min:sec**
ABIDEII-BNI_1	9	900	3.10	6.7	240.5	2,500	SS1.8	—	S	AP	1.06	1.06	1.06	256	256	193	5:34
ABIDEII-EMC_1	16	350	4.24	10.26	81.4	1,664	AP2	—	S	AP	0.90	0.90	0.90	256	256	186	5:40
ABIDEII-ETH_1	8	1,150	3.90	7.9	188.3	3,000	SP2.3	—	T	RL	0.90	0.90	0.90	256	256	180	5:46
ABIDEII-GU_1	7	1,100	3.50	8.2	190	2,530	GP2	—	S	AP	1.00	1.00	1.00	256	256	276	8:05
ABIDEII-IU_1	8	1,000	2.30	7	210	2,400	GP2	P×7/8	S	AP	0.70	0.70	0.70	320	320	256	7:02
ABIDEII-IP_1	30	–	5.60	25	141.7	2,500	SP2+SS2	—	S	AP	1.00	1.00	1.00	240	240	170	4:37
ABIDEII-KKI_1* ^(8 channels)^	8	1,000	3.70	8	191.5	3,500	SS2	—	C	RL	1.00	1.00	1.00	256	200	200	8:08
ABIDEII-KKI_1* ^(32 channels)^	8	900	3.70	8.2	192.9	3,000	SP1.2+SS2	—	T	RL	0.95	0.95	1.00	224	224	150	4:24
ABIDEII-KUL_3	8	900	4.60	9.4	130.6	2,000	SP1.5+SS2.5	—	C	RL	0.98	0.98	1.20	256	256	182	1:43
ABIDEII-NYU_1	7	1,100	3.25	7.4	200	2,530	—	—	S	AP	1.30	1.00	1.33	256	256	128	8:07
ABIDEII-NYU_2	7	1,100	3.25	7.2	200	2,530	—	—	S	AP	1.30	1.00	1.33	256	256	128	8:07
ABIDEII-ONRC_2	13	794	2.88	7.1	200	2,200	GP3	—	T	RL/AP†	0.80	0.80	0.80	220	320	208	3:25
ABIDEII-OHSU_1	10	900	3.58	8.2	180	2,300	—	—	S	AP	1.00	1.00	1.10	256	240	160	9:14
ABIDEII-SDSU_1	8	600	3.17	8.136	244.1	2,683‡	—	—	S	AP	1.00	1.00	1.00	256	256	172	4:54
ABIDEII-TCD_1	8	1,150	3.90	7.9	188.3	3,000	SP2.3	—	T	RL	0.90	0.90	0.90	256	256	180	5:43
ABIDEII-UCD_1	8	1,050	3.16	7.5	220	2,000	GP2	—	S	AP	1.00	1.00	1.00	256	224	192	4:06
ABIDEII-UCLA_1	9	853	2.86	6.7	240	2,300	—	—	S	AP	1.00	1.00	1.20	256	240	160	9:14
ABIDEII-USM_1	9	900	2.91	6.8	240	2,300	—	—	S	AP	1.00	1.00	1.20	256	240	160	9:14
ABIDEII-UCLA_Long	9	853	2.86	6.7	240	2,300	—	—	S	AP	1.00	1.00	1.20	256	240	160	9:14
ABIDEII-UPSM_Long	7	1,000	3.93	9.4	130	2,100	—		S	AP	1.05	1.05	1.05	256	256	176	8:59
The 3D MPRAGE (three dimensional magnetization prepared rapid acquisition gradient echo) sequence, or a vendor specific variant, was used to acquire all data. BW, bandwidth per pixel; Dims, dimensions; ES, echo spacing; FA, flip angle (indexed in degrees); PA, parallel acquisition; PE, phase encoding; PF, partial Fourier (halfscan); SO, Slice orientation; TA, Acquisition Time; TE, echo time; TI, inversion time; TR, repetition time; For parallel acquisitions; SS, SENSE acceleration in the slice direction; SP, SENSE acceleration in the phase encoding direction; GP, GRAPPA acceleration in the phase encoding direction; AP, ASSET acceleration in the phase encoding direction. For partial Fourier, the under-sampled dimension is listed with the under sampling factor; P, phase encoding. For slice orientation; S, sagittal; T, transverse (axial); C, coronal. For phase encoding direction; RL, right-to-left; AP, Anterior to posterior. Reconstructed resolution and image dimensions refer to the images after they have been reconstructed from the k-space data, the matrix size and resolution used for the acquisition may differ. For these categories, RO, read out direction; PE, phase encoding direction, and SL, slice direction.																	

*62 datasets of the KKI-1 collection were acquired with an 8-channel head coil, 149 datasets were acquired with a 32 channel coil and different scanning parameters. A list of the IDs with the corresponding head coil is provided in the ABIDE II website page for this collection as ‘Additional scan Information.’

^†^ ONRC_2 has variable phase encoding direction, specific information for each dataset is provided in the release/website (http://fcon_1000.projects.nitrc.org/indi/abide/).

^‡^ SDSU has used a GE scanner that refers to TR as the amount of time between the RF pulses that are used to read out the lines of k-space (i.e., echo space). Here to be consistent across all collections we report TR using the Siemens convention that refers to TR as the time between sequential inversion recovery pulses. Based on the simple equation TR=TI+n*ES, where n is the number of actual (corrected for parallel imaging) phase encoding lines, we calculated the TR of SDSU to be 2,683 ms.

**Table 3:** Sequence parameters of resting state fMRI datasets at each data collection included in the primary ABIDE II.

**Collection**	**FA**	**TE**	**TR**	**BW**	**PA**	**PF**	**PE**	**FS**	**SO**	**SA**	**Gap**	**Recon resolution (mm)**			**Recon image Matrix (px)**			**Nacq**	**Ndisc**	**TA**
**Label**	**°**	**ms**	**ms**	**Hz/Px**							**%**	**RO**	**PE**	**SL**	**RO**	**PH**	**SL**			**min:sec**
ABIDEII-BNI_1	80	25	3,000	3,280	SP2	—	AP	N	T	A	0	3.75	3.75	4.00	64	64	50	120	0	6:09
ABIDEII-EMC_1	85	30	2,000	7,812	—	—	AP		T	ID	0	3.59	3.59	4.00	64	64	37	160		5:20
ABIDEII-ETH_1	90	25	2,000	1,590	SP2.5	—	AP	Y	T	D	10	3.00	3.00	3.30	80	80	40	210	0	7:06
ABIDEII-GU_1	90	30	2,000	2,442	GP2	—	AP	N	T	IA	20	3.00	3.00	3.00	64	64	43	154*	2	5:14
ABIDEII-IU_1	60	28	813	2,604	MB3	—	AP	Y	TO	IA	0	3.44	3.44	3.40	64	64	42	433	2	6:00
ABIDEII-IP_1	90	45	2,700	2,213	—	—	AP	Y	T	A	0	3.60	3.70	4.00	64	64	32	85	2	7:55
ABIDEII-KKI_1†	75	30	2,500	2,697	SP3	—	AP	Y	T	A	0	3.00	3.00	3.00	96	96	47	156	2	6:40
ABIDEII-KUL_3	90	30	2,500	2,188	SP2	—	AP	Y	T	A	14.8	1.56	1.56	3.10	128	128	45	162	4	7:00
ABIDEII-NYU_1	90	15	2,000	3,906	—	—	RL	Y	TO	IA	0	3.00	3.00	4.00	80	64	33	180	2	6:00
ABIDEII-NYU_2	82	30	2,000	3,906	—	—	RL	Y	T	IA	0	3.00	3.00	3.00	80	64	34	180	2	6:00
ABIDEII-OHSU_1	90	30	2,500	2,298	—	—	AP	Y	TO	IA	0	3.75	3.75	3.80	64	64	36	120	2	5:07
ABIDEII-ONRC_2	60	30	475	2,604	MB8	—	AP	Y	T	IA	0	3.00	3.00	3.00	80	80	48	947	2	7:37
ABIDEII-SDSU_1	90	30	2,000	7,813	A	—	AP	N	T	IA	0	3.44	3.44	3.40	64	64	42	180	5	6:10
ABIDEII-TCD_1	90	27	2,000	2,420	—	—	AP	Y	T	A	10.94	3.00	3.00	3.20	80	80	37	210	0	7:06
ABIDEII-UCD_1‡	90	24	2,000	2,232	—	—	AP	Y	TO	IA	0	3.50	3.50	3.50	64	64	36	151	2	5:06
ABIDEII-UCLA_1	90	28	3,000	2,442	—	—	AP	Y	TO	IA	0	3.00	3.00	4.00	64	64	34	120	2	6:06
ABIDEII-USM_1	90	28	2,000	2,894	GP2	—	AP	Y	TO	IA	10	3.40	3.40	3.00	64	64	40	240	2	8:06
ABIDEII-UCLA_Long	90	28	3,000	2,442	—	—	AP	Y	TO	IA	0	3.00	3.00	4.00	64	64	34	120	2	6:06
ABIDEII-UPSM_Long	70	25	1,500	3,126	—	—	AP	Y	TO	IA	0	3.13	3.13	4.00	64	64	29	200	2	5:06
All data were collected with echo planar imaging (EPI) sequences. BW, bandwidth per pixel; Dims, dimensions; FA, flip angle; FS, fat suppression; Gap, gap between slices; Nacq, number of volumes collected; Ndisc, number of initial volumes discarded by the scanner; PA, parallel acquisition; PE, Phase encoding; PF, Partial Fourier (half scan); SA, slice acquisition order; SO, slice orientation; TA, acquisition time; TE, echo time; TR, repetition time. For parallel acquisition the acceleration technology and dimension are listed followed by the acceleration factor, AP, ASSET acceleration in the phase encoding direction; GP, GRAPPA acceleration in the phase encoding direction; MB, multi-band imaging; SP, SENSE acceleration in the phase encoding direction. For partial Fourier, the under-sampled dimension is listed with the under sampling factor, P, phase encoding. For slice orientation; T, transverse (axial), and TO, transverse oblique. For phase encoding direction; RL, right-to-left; AP, Anterior to posterior. For slice acquisition order; A, ascending; D, descending; IA, interleaved ascending and ID, interleaved descending. Reconstructed resolution and image dimensions refer to the images after they have been reconstructed from the k-space data, the matrix size and resolution used for the acquisition may differ. For these categories, RO, read out direction; PE, phase encoding direction, and SL, slice direction.																				

*GU discarded the first 2 scans in addition to the 2 discarded by the sequence resulting in 152 volumes.

^†^ For the KKI_1 collection, an 8-channel head coil was used for *n*=149 datasets and a 32-channel head coil was used for *n*=62 datasets—see Table 1.

^‡^ One R-fMRI datasets was collected with different EPI sequence with voxel size 3.5×3.5×4—specifics are provided in the ABIDE II website (http://fcon_1000.projects.nitrc.org/indi/abide/).

**Table 4:** Sequence parameters of diffusion MRI datasets for each of the six collections sharing these data along with corresponding high resolution anatomical and resting state functional MRI data.

**Collection**	**TE**	**TR**	**BW**	**FS**	**PA**	**PF**	**PE**	**SO**	**Gap**	**Recon Resolution (mm)**			**Recon Image Matrix (px)**			**Nb0**	**Ndir**	**Bvals**	**Navg**	**TA**
**Label**	**ms**	**ms**	**Hz/Px**						**%**	**RO**	**PE**	**SL**	**RO**	**PH**	**SL**					**min:sec**
ABIDEII-BNI_1	101	7,850	2,621.1	Y	SP2	None	AP	T	0	1.41	1.41	3	192	192	48	1	32	2,500	1	4:34
ABIDEII-IP_1	86	5,407	1,972.5	Y	SP2	P×0.683	AP	T	0	2.5	2.5	2.5	96	96	45	1	32	1,000	1	3:09
ABIDEII-NYU_1	78	5,200	3,720	Y	None	None	RL	T	0	3	3	3	64	64	50	1	64	1,000	1	5:43
ABIDEII-NYU_2																				
ABIDEII-SDSU_1*	81.8	8,500	3,906.25	Y	None	None	RL	T	0	0.94	0.94	2	256	256	68	1	61	1,000	1	8:56
	7.5	1,097	250	Y	None	None	RL	T	0	1.88	1.88	2	128	128						5:34
ABIDEII-TCD_1	79	20,244	2,590.6	Y	SP2	None	AP	T	0	1.94	1.94	2	128	128	65	1	61	1,500	4	24:21
All diffusion data were collected with spin echo planar imaging (SE-EPI) sequences. *Data were collected with two slightly different sequences; Bvals, the gradient strength used for diffusion weighting; BW, bandwidth per pixel; Dims, dimensions; FS, fat suppression; Gap, gap between slices; Navg, number of volumes collected for each direction and subsequently averaged; Nb0, number of volumes acquired with b=0; Ndir, number of directions acquired with diffusion weighting; PA, parallel acquisition; PE, Phase encoding; PF, Partial Fourier (half scan), SO, Slice orientation; TA, Acqusition Time; TE, echo time; TR, repetition time. For parallel acquisition the acceleration technology and dimension are listed followed by the acceleration factor, SP, SENSE acceleration in the phase encoding direction. For partial Fourier, the under-sampled dimension is listed with the undersampling factor, P, phase encoding. For slice orientation; T, transverse (axial). For phase encoding direction; RL, right-to-left; AP, Anterior to posterior. Reconstructed resolution and image dimensions refer to the images after they have been reconstructed from the k-space data, the matrix size and resolution used for the acquisition may differ. For these categories, RO, read out direction; PE, phase encoding direction, and SL, slice direction.																				

**Table 5:** Spatial and temporal indices of MRI data quality selected from the Preprocessed Connectome Project.

**Spatial Metrics**	**Description**
Contrast-to-noise ratio (CNR)^50^ (sMRI only)	M_GM_ intensity—M_WM_ intensity/SD_air_ intensity. *Larger values reflect a better WM GM distinction.*
Signal-to-noise ratio (SNR)^50^	M_GM_ intensity/SD_air_ intensity. *Larger values reflect less noise*
Artifactual voxel detection (Qi1)^51^ (sMRI only)	* voxels with intensity corrupted by artifacts/ *voxels in the background. *Larger values reflect more artifacts which likley due to motion or image instability.*
Entropy Focus Criteria (EFC)^65,^ †	Shannon’s entropy of each voxel's intensity used to measure ghosting and blurring due to head motion. *Larger values reflect more blurring likley to motion or techincal differences.*
Smoothness of Voxels^62^ (FWHM)†	Full-width half maximum of the spatial distribution of the image intensity values. *Larger values reflect more spatial smoothing maybe due to motion or technical differences.*
Foreground to Background Energy Ratio (FBER)†	M energy of image intensity (i.e., mean of squares) within the head relative to that of outside the head. *Larger values reflect higher signal in relation to noise.*
Ghost to Signal Ratio (GSR)^66,^ †	M signal in the ‘ghost’ image divided by the M signal within the brain. *Larger values reflect more ghosting likley due to physiological noise, motion, or technical issues.*
	
Temporal Metrics (R-fMRI* and DTI only)	
Mean framewise displacement- Jenkinson (mFD)^52,^ ‡	Sum absolute displacement changes in the x, y and z directions and rotational changes around them. Rotational changes are given distance values based on changes across the surface of a 50 mm radius sphere. *Larger values reflect more movement.*
% and * volumes with FD>0.2 mm‡	% and *volume to volume motion >0.2 mm FD. *Larger values reflect more movement.*
Standardized DVARS^63,^ ‡	Spatial SD of the data temporal derivative normalized by the temporal SD and autocorrelation. *Larger values reflect larger frame-to-frame differences in signal intensity due to head motion or scanner instability.*
Outlier Detection^67,^ †	M fraction of outliers in each volume per 3dToutcount AFNI command. *Higher values reflect more outlying voxels, which may be due to scanner instability or RF artifacts.*
Global Correlation (GCORR)^64,^ ‡	M correlation of all combinations of voxels in a time series. Illustrates differences between data due to motion/physiological noise. *Larger values reflect a greater degree of spatial correlation between slices, which may be due to head motion or ‘signal leakage’ in simultaneous multi-slice acquisitions.*
Median Distance Index^67,^ ‡	M distance (1—spearman’s rho) between each time-point's volume and the median volume using AFNI’s **3dTqual** command. *Higher values reflect greater differences between subsequent frames, which may be due to head motion or technical issues.*
They have been computed for all structural MRI (sMRI), Diffusion Tensor Imaging (DTI), and Resting State functional MRI (R-fMRI) datasets unless indicated otherwise. All are released for each dataset in ABIDE II (file and link of release pending). See Figs 3 and 4 and Supplementary Fig. 1 for illustrations of the distribution of a selection of spatial and temporal metrics within and across collection.	

*For all R-fMRI data temporal metrics have been computed after discarding the first 5 time points of the time series which were field map corrected if field maps were provided (only in the SDSU_1 data collection). Computation of all spatial metrics excluded absolute zero background values.

^†^ For R-fMRI data these metrics are computed on mean functional data.

^‡^ For R-fMRI these metrics are computed on time series data. M, Mean; GM, Gray Matter; WM, White Matter; s.d., Standard Deviation.
