# Supplementary material for: Enhancing studies of the connectome in autism using the autism brain imaging data exchange II
Source: Sci Data. 2017 Mar 14;4:170010. doi: 10.1038/sdata.2017.10 (PMC5349246; doi:10.1038/sdata.2017.10)
Supplement: Supplementary Information [file sdata201710-s2.pdf]

Supplementary Information

Enhancing studies of the connectome in autism using the Autism Brain Imaging Data Exchange II

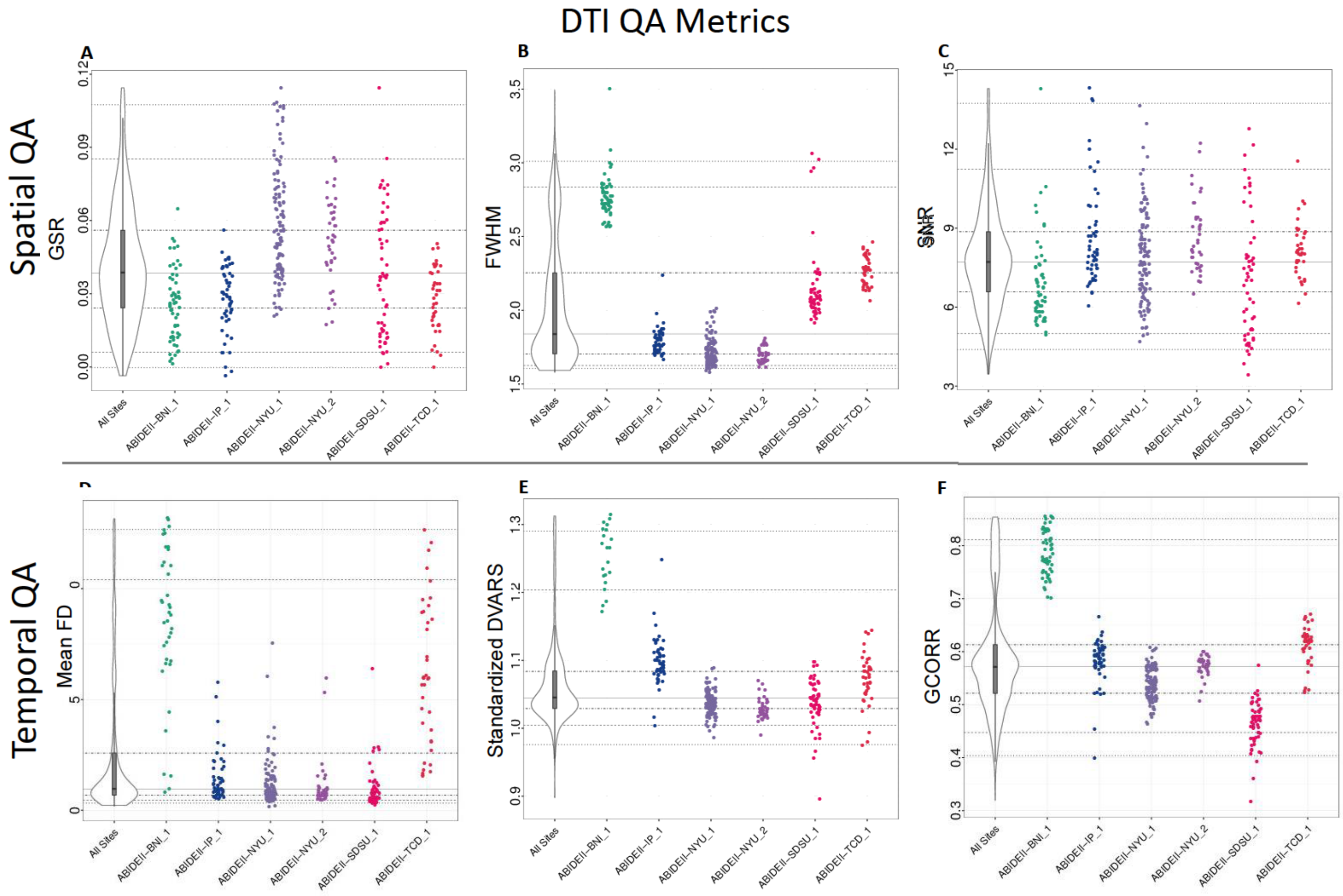

Supplementary Figure 1. Selection of spatial and temporal quality metrics for the diffusion weighted imaging released for six data collections of ABIDE II. Spatial metrics include: (**A**) Ghost to single ratio (GSR)[64]; (**B**) smoothness of voxels indexed as full-width half maximum (FWHM)[63], (**C**) signal-to-noise ratio (SNR)[50]. Temporal metrics are: (**D**) mean framewise displacement[52]; standardized DVARS[65], and global correlation (GCORR)[67] - See Table 5 for details on this and the other quality metrics released. The colored scatterplots illustrate the quality metrics distribution for spatial MRI dataset within a given ADBIE II collection (17 cross-sectional and 2 longitudinal collections). The black and white violin plots represent a kernel density estimation of the distribution across all datasets for each quality metrics with its midline thick gray line representing the value that occurs most commonly in the

distribution. For each plot, the horizontal gray lines mark the 1st, 5th, 25th, 50th (solid gray line), 75th, 95th and 99th percentiles starting from the bottom.

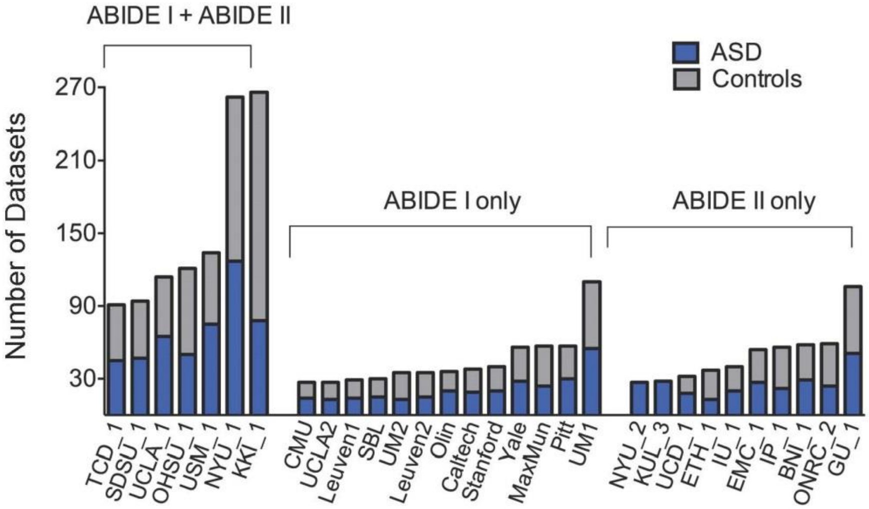

Supplementary Figure 2. Illustration of individual cross-sectional data collections available across ABIDE I and II (N=27 collections, N=2156 datasets across 23 institutions). Seven collections in ABIDE II represents additions of unique datasets to collections originally shared in ABIDE I; their combination yields a common multisite cross-sectional aggregate of 1082 datasets (n=487 ASD; n=595 controls). The remaining collections are independent between the two ABIDE initiatives and include 13 ABIDE I cross-sectional collections (n=280 ASD; n=297 controls) and seven new cross-sectional collections in ABIDE II (n=259 ASD; n=238 controls). These datasets can be combined or split for large-scale discovery and/or replication efforts.

Collection Label	Contributing Institutions	Principal Investigator(s) and Key Contributor(s)
ABIDEII-BNI_1	Barrow Neurological Institute	Leslie Baxter, B Blair Braden, Christopher Smith
ABIDEII-EMC_1	Erasmus University Medical Center Rotterdam	Tonya White, Laura Blanken, Ryan Muetzel, Henning Tiemeier, Frank Verhulst
ABIDEII-ETH_1	ETH Zurich	Nicole Wenderoth, Joshua Balsters
ABIDEII-GU_1	Georgetown University	Chandan Vaidya, Lauren Kenworthy
ABIDEII-IU_1	Indiana University	Daniel Kennedy, Lisa Byrge
ABIDEII-IP_1	Institute Pasteur & Robert Debré Hospital	Richard Delorme, Anita Beggiato, Roberto Toro, Monique Elmaleh, Marianne Alison, David Germanaud
ABIDEII-KKI_1	Kennedy Krieger Institute	Stewart Mostofsky, Mary Beth Nebel
ABIDEII-KUL_3	KU Leuven	Kaat Alaerts, Claudia Dillen, Sylvie Bernaerts, Jellina Prinsen
ABIDEII-NYU_1	NYU Langone Medical Center: Sample 1	Adriana Di Martino, F. X. Castellanos, Michael P Milham, Clare Kelly
ABIDEII-NYU_2	NYU Langone Medical Center: Sample 2	Adriana Di Martino, F. X. Castellanos, Michael P Milham
ABIDEII-ONRC_2	Olin Institute of Living Hartford	Michal Assaf
ABIDEII-OHSU_1	Oregon Health and Science Univeristy	Damien Fair, Joel Nigg, Eric Fombonne
ABIDEII-SDSU_1	San Diego State University	Ralph-Axel Müller, Inna Fishman, R Joanne Jao Keehn
ABIDEII-TCD_1	Trinity Centre for Health and Sciences	Louise Gallagher, Jane McGrath, Jacqueline Fitzgerald
ABIDEII-UCD_1	University of California Davis	Marjorie Solomon
ABIDEII-UCLA_1	University of California Los Angeles	Mirella Dapretto, Susan Bookheimer
ABIDEII-USM_1	University of Utah School of Medicine	Jeffrey Anderson, Jared Nielsen, Alyson Froehlich, Molly DuBray, Michael Ferguson, Jason Druzgal, Annahir Cariello, Jason Cooperrider, Brandon Zielinski, Caitlin Ravichandran, Thomas Fletcher, Andrew Alexander, Erin Bigler, Nicholas Lange, Janet Lainhart
UPSM_Long	Univeristy of Pittsburgh	Kirsten O'Hearn, Beatriz Luna
UCLA_Long	University of California Los Angeles	Mirella Dapretto, Susan Y. Bookheimer

Supplementary Table 1: Information on the institutions contributing to ABIDE II primary data collections (i.e., one time point datasets collected from individuals not previously included in ABIDE I) and longitudinal collections (i.e., providing longitudinal MRI data of individuals contributed in ABIDE I).

Data collection	Phenotype														
Code	IQ*		Handedness		**ADOS*[15,16]**		**ADI-R[17]**		**SRS[18]**		**RBS-R[19,20]**		**ASD Comorbidity**	Meds Info		Other
	ASD	*Contr*	*ASD*	*Contr*	*ASD*	*Contr*	*ASD*	*Contr*	*ASD*	*Contr*	*ASD*	*Contr*	*ASD*	*ASD*	*Contr*	
ABIDEII-BNI_1	29	29	29	29	29	0	0	0	28	29	20	0	0	29	29	*BDI*[1]
ABIDEII-EMC_1	24	25	27	27	0	0	0	0	0	0	0	0	27	27	27	-
ABIDEII-ETH_1	13	24	13	24	12	0	12	0	11	22	0	0	0	0	0	SCQ[2]
ABIDEII-GU_1	50	54	51	55	51	0	49	0	51	55	0	0	0	51	55	*BRIEF*[3], *CASI*[4], *CBCL*[5], *CSI*[6], *MASC*[7]
ABIDEII-IU_1	20	20	20	20	20	0	0	0	15	11	0	0	0	0	0	AQ[8], *BDI*[1]
ABIDEII-IP_1	22	34	22	34	22	0	12	0	0	0	14	19	22	22	34	-
ABIDEII-KKI_1	56	155	56	155	56	0	55	0	52	148	51	139	56	53	152	*BASC*[9], *BRIEF*[3,] *CBCL*[5]
ABIDEII-KUL_3***	28	0	28	0	28	0	0	0	28	0	27	0	27	28	0	-
ABIDEII-NYU_1	47	30	37	29	48	0	45	0	48	28	9	11	48	48	30	AQ[8], *BRIEF*[3], *CBCL*[5,] *CPRS-R:LV*[10], *MASC*[7], SCQ[2], VABS-II[11], WIAT[12]
ABIDEII-NYU_2***	27	0	25	0	27	0	27	0	27	0	24	0	27	27	0	*BRIEF*[3], *CBCL*[5], *CPRS-R;LS*[10], *MASC*[7], SCQ[2], VABS-II[11], WIAT[12]
ABIDEII-ONRC_2	24	35	24	35	23	35	11	0	0	0	0	0	24	24	35	*BDI*[1]
ABIDEII-OHSU_1	37	56	37	56	37	2	37	0	33	46	0	0	32	37	56	*MASC*[7], SCQ[2]
ABIDEII-SDSU_1	33	25	33	24	33	0	33	0	32	25	33	25	0	33	25	*BRIEF*[2], CELF[13], SCQ[2], VMI[14]
ABIDEII-TCD_1	21	21	21	21	21	0	21	0	21	21	21	0	0	21	21	*CBCL*[5], SCQ[2]
ABIDEII-UCD_1	18	14	18	14	18	0	0	0	18	14	18	14	18	18	14	*BASC*[9], SCQ[2]
ABIDEII-UCLA_1	16	14	16	16	16	0	0	0	0	0	0	0	0	16	14	-
ABIDEII-USM_1	17	15	15	10	12	11	0	0	14	8	0	0	0	14	7	-
	**482**	**551**	**472**	**549**	**453**	**48**	**302**	**0**	**378**	**407**	**217**	**208**	**281**	**448**	**499**	-

Supplementary Table 2: Counts of available phenotypic measures in each primary ABIDE II data collection (i.e., collections from individuals not included in ABIDE I). Counts are only for measures shared across at least 50% (n=8) of the collections; the remaining measures available are listed in the column labeled Other (measures of psychopathology are in italic). Age and sex are available for all data available, plotted in Figure 1 and summarized by diagnostic group in Supplementary Table 4. *Any IQ indices (i.e., full, verbal or performance IQ; data records provide information on the standard IQ test utilized); **: 8 data collections shared the Autism Diagnostic Observation Schedule - Second Edition (ADOS-2) calibrated severity scores (see Figure 1E); 14 collections shared the ADOS-G scaled scores. *** These data collections include only data from individuals with ASD. ADI-R: Autism Diagnostic

Interview-Revised; ASD: Autism Spectrum Disorder; AQ: Autism Quotient; BASC: Behavior Assessment System for Children; BDI: Beck Depression Inventory; BRIEF: Behavior Rating Inventory of Executive Function; CASI: Child and Adolescent Symptom Inventory; CBCL: Child Behavior Checklist; CELF: Clinical Evaluation of Language Fundamentals; CSI: Child Symptom Inventory; CPRS-R:LV: Conners' Parent Rating Scale-Revised; Long Version; MASC: Multidimensional Anxiety Scale for Children; Meds info: use of medication within three months from scan; RBS-R: Repetitive Behavior Scale Revised; SCQ: Social Communication Questionnaire; SRS: Social Responsiveness Scale; VABS-II: Vineland Adaptive Behavior Scale, Second Edition; VMI: Beery-Buktenica Developmental Test of Visual-Motor Integration; WIAT: Wechsler Individual Achievement Test. See Supplemntary text for reference of the instruments listed here.

Collection code	N		Females n (%)		Age Range M ± SD				*IQ Range M ± SD				Handedness n R:L:M	
	ASD	*TC*	*ASD*	*TC*	*ASD*		*TC*		*ASD*		*TC*		*ASD*	*TC*
ABIDEII-BNI_1	29	29	0	0	18-62	38 ± 16	18-64	40 ± 15	85-139	108 ± 13	89-141	112 ± 12	29:0:0	29:0:0
ABIDEII-EMC_1	27	27	5 (19)	5 (19)	6-11	8 ± 1	6-10	8 ± 1	67-122	98±15	71-135	99±16	22:5:0	21:6:0
ABIDEII-ETH_1	13	24	0	0	15-27	20 ± 3	14-31	24 ± 4	82-123	109 ± 13	100-133	117 ± 9	13:0:0	13:0:0
ABIDEII-GU_1	51	55	8 (16)	27 (49)	8-14	11 ± 2	8-14	10 ± 2	90-149	118 ± 15	91-149	121 ± 14	43:8:0	52:3:0
ABIDEII-IU_1	20	20	4 (20)	5 (25)	17-54	25 ± 9	19-37	24 ± 5	80-132	116 ± 12	94-135	117 ± 10	15:2:3	17:1:2
ABIDEII-IP_1	22	34	8 (36)	22 (65)	6-27	15 ± 5	8-47	23 ± 12	49-125	92 ± 24	71-125	106 ± 17	21:1:0	27:5:2
ABIDEII-KKI_1	56	155	15 (27)	56 (36)	8-13	10 ± 2	8-13	10 ± 1	63-132	103 ± 16	85-143	114 ± 11	46:2:8	133:10:12
ABIDEII-KUL_3**	28	0	0	0	18-35	24 ± 5	NA	NA	73-146	107 ± 16	NA	NA	22:6:0	NA
ABIDEII-NYU_1	48	30	5 (10)	2 (7)	5-35	10 ± 6	6-24	10 ± 3	67-138	102 ± 18	91-144	116 ± 15	27:2:8	28:0:1
ABIDEII-NYU_2**	27	0	3 (11)	0	5-9	7 ± 1	NA	NA	82-143	107 ± 14	NA	NA	14:4:7	NA
ABIDEII-ONRC_2	24	35	4 (17)	15 (43)	18-31	22 ± 4	18-30	24 ± 4	80-146	114 ± 16	85-146	111 ± 13	16:4:4	32:0:3
ABIDEII-OHSU_1	37	56	7 (19)	29 (52)	7-15	12 ± 2	8-14	10 ± 2	72-136	106 ± 17	90-140	117 ± 12	35:1:1	55:0:1
ABIDEII-SDSU_1	33	25	7 (21)	2 (8)	7-18	13 ± 3	8-18	13 ± 3	66-130	100 ± 15	79-125	103 ± 11	27:4:2	20:1:3
ABIDEII-TCD_1	21	21	0	0	10-20	15 ± 3	10-20	16 ± 3	79-139	109 ± 15	81-142	118 ± 13	21:0:0	21:0:0
ABIDEII-UCD_1	18	14	4 (22)	4 (28)	12-18	15 ± 2	12-17	15 ± 2	83-130	103 ± 12	92-128	113 ± 11	17:0:1	14:0:0
ABIDEII-UCLA_1	16	16	1 (6)	5 (31)	8-15	12± 2	8-14	10 ± 2	78-124	102 ± 14	94-141	115 ± 13	14:2:0	14:1:1
ABIDEII-USM_1	17	16	2 (12)	3 (19)	9-39	18 ± 7	12-36	24 ± 8	73-134	99 ± 19	86-144	115 ± 16	13:0:2	9:0:1
	**487**	**557**	**73 (15)**	**175 (31)**	**5-62**	**15 ± 9**	**6-64**	**15 ± 10**	**49-149**	**107 ± 17**	**71-149**	**115 ± 13**	**395:41:36**	**485:27:26**

Supplementary Table 3: Basic statistics (i.e., minimum, maximum, mean and standard deviation) of key phenotypic information in each primary ABIDE II data collection by diagnostic group. *: Full IQ is reported for all collections but EMC_1 for which Performance IQ scores are available. **: These data collections include only data from individuals with ASD. ASD: Autism Spectrum Disorder; Handedness R: Right handed (defined as primarily using their right hand to perform all tasks; scores ≥ +50); L: Left handed (defined as primarily using their left hand to perform all tasks; scores ≤ -50); M: Mixed handed (change of hand preferences in different tasks; scores between -50 and 50); M ± SD: Mean ± Standard Deviation; NA: not applicable.

Supplementary Material Citations

1 Beck, A. T., Steer, R. A. & Brown, G. K. *Beck depression inventory-II*. (Psychological Corporation, 1996).
2 Rutter, M., Bailey, A. & Lord, C. *The social communication questionnaire: Manual*. (Western Psychological Services, 2003).
3 Achenbach, T. & Rescorla, L. *Manual for the ASEBA school-age forms & profiles: an integrated system of multi-informant assessment* (University of Vermont. Research Center for Children, Youth, & Families, 2001).
4 Gadow, K. & Sprafkin, J. *Child and adolescent symptom inventory-4R*. (Checkmate Plus, 2005).
5 Achenbach, T. & Rescorla, L. *Manual for the ASEBA school-age forms & profiles: an integrated system of multi-informant assessment* (University of Vermont. Research Center for Children, Youth, & Families, 2001).
6 Gadow, K. D. & Sprafkin, J. N. *Child symptom inventory 4: Screening and norms manual*. (Checkmate Plus, 2002).
7 March, J. S., Parker, J. D., Sullivan, K., Stallings, P. & Conners, C. K. The Multidimensional Anxiety Scale for Children (MASC): factor structure, reliability, and validity. *Journal of the American Academy of Child and Adolescent Psychiatry* **36**, 554-565, doi:10.1097/00004583-199704000-00019 (1997).
8 Baron-Cohen, S., Wheelwright, S., Skinner, R., Martin, J. & Clubley, E. The Autism-Spectrum Quotient (AQ): Evidence from Asperger Syndrome/High-Functioning Autism, Malesand Females, Scientists and Mathematicians. *Journal of autism and developmental disorders* **31**, 5-17, doi:10.1023/a:1005653411471 (2001).
9 Reynolds, C. R. & Kamphaus, R. W. *BASC-2: Behavior assessment system for children*. (2004).
10 Conners, C. K. *Conners' rating scales revised*. (Multi-Health Systems, Incorporated, 2001).
11 Sparrow, S. S., Cicchetti, D. V. & Balla, D. A. *Vineland II: Vineland Adaptive Behavior Scales Second Edition*. (Pearson Assessments, 2005).
12 Wechsler, D. *Wechsler Individual Achievement Test Second Edition Abbreviated (WIAT-II Abbreviated)*. (The Psychological Corporation, 2005).
13 Oldfield, R. C. The assessment and analysis of handedness: the Edinburgh inventory. *Neuropsychologia* **9**, 97-113 (1971).
14 Beery, K. E., & Beery, N. A. *Beery-Buktenica Developmental Test of Visual-Motor Integration - Sixth Edition*. (NCS Pearson, Inc, 2010).
15 Gotham, K., Risi, S., Pickles, A. & Lord, C. The Autism Diagnostic Observation Schedule: revised algorithms for improved diagnostic validity. *Journal of autism and developmental disorders* **37**, 613-627, doi:10.1007/s10803-006-0280-1 (2007).
16 Lord, C., Rutter, M., DiLavore, P. C. & Risi, S. *Autism diagnostic observation schedule*. (Western Psychological Services, 1999).
17 Lord, C., Rutter, M. & Le Couteur, A. Autism Diagnostic Interview-Revised: a revised version of a diagnostic interview for caregivers of individuals with possible pervasive developmental disorders. *Journal of autism and developmental disorders* **24**, 659-685 (1994).
18 Constantino, J. N. & Gruber, C. P. *Social responsiveness scale (SRS): Manual*. (Western Psychological Services, 2007).
19 Bodfish, J. W., Symons, F. J., Parker, D. E. & Lewis, M. H. Varieties of repetitive behavior in autism: comparisons to mental retardation. *Journal of autism and developmental disorders* **30**, 237-243 (2000).
20 Lam, K. S. & Aman, M. G. The Repetitive Behavior Scale-Revised: independent validation in individuals with autism spectrum disorders. *Journal of autism and developmental disorders* **37**, 855-866, doi:10.1007/s10803-006-0213-z (2007).
